# A case study of the Ancientbiotics collaboration

**DOI:** 10.1016/j.patter.2022.100632

**Published:** 2022-12-09

**Authors:** Erin Connelly, Christina Lee, Jessica Furner-Pardoe, Charo I. del Genio, Freya Harrison

**Affiliations:** 1 School of Life Sciences, Gibbet Hill Campus, University of Warwick, Coventry CV4 7AL, UK; 2 School of English, University Park, University of Nottingham, Nottingham NG7 2RD, UK; 3 Warwick Medical School, Gibbet Hill Campus, University of Warwick, Coventry CV4 7AL, UK; 4 Centre for Fluid and Complex Systems, Coventry University, Coventry CV1 5FB, UK

**Keywords:** interdisciplinary, collaboration, history of medicine, medieval studies, ethnopharmacology, datamining, network analysis, AMR

## Abstract

Interdisciplinary collaboration is regarded as a desirable way of researching and, in some instances, even a requirement for academic teams and funding proposals. This paper explores the possibilities, but also the problems, of collaboration between different disciplines through a case study of the Ancientbiotics team. This team explores the potential of natural products contained in historical medical recipes. The search for clinically useful natural products in unusual places, such as historical medical practices, is a well-established endeavor in the scientific disciplines. The Ancientbiotics collaboration, largely based across UK institutions, takes this path a step forward in combining modern scientific knowledge of natural products with expertise from humanities to identify ingredient combinations. After 7 years of practice, the research has produced a variety of outcomes. This perspective will explore how the team worked within an interdisciplinary framework to advance investigation and application of historical medical recipes.

## The bigger picture

Collaborations that cross traditional boundaries between disciplines in STEM and the arts and humanities open up exciting research possibilities. In our team’s case, we combined expertise in historical manuscripts, data science, and microbiology to explore the structure and potential efficacy of historical medical recipes. Such an approach can highlight patterns or questions that a single-disciplinary approach is likely to miss. But learning to speak each other’s disciplinary languages is not always easy, and misunderstandings can impede work. Here, we present our own experiences as a case study of how we have learned from each other to ask new questions of our source material and the problems we have had to solve along the way.

## Teaser

How can data scientists, humanities researchers, and bench scientists join forces to explore interdisciplinary research questions? Researchers from different backgrounds may view the same dataset in very different ways, and while combining these diverse approaches can pay dividends, it is not always easy. We present our experience of the payoffs and pitfalls of conducting interdisciplinary research into the knowledge contained in historical medical texts in the hope that our story can aid others in team-building across disciplines.

## Introduction

The Ancientbiotics team shares two objectives. First, we aim to use interdisciplinary approaches to examine the construction of medieval infection remedies and the knowledge transmission processes behind them. Second, we aim to identify potential clinically useful natural products inspired by the remedies of historical and traditional sources, such as medieval texts.^1^ The team developed out of a pilot study on a 1,000-year-old early medieval remedy for suspected eye infection (“Bald's Eyesalve”) with the support of a University of Nottingham Interdisciplinary Centre for Analytical Science sandpit grant for interdisciplinary research and a crowdfunding campaign.^2^ The initial team consisted of a small group of researchers from two departments (School of English and Centre for Biomolecular Sciences) at the University of Nottingham, with shared interests in medieval studies and natural product potential for antimicrobial activity. It has since expanded to a multiinstitutional, international, and multidisciplinary consortium. The collaboration has created research projects for a number of students from the undergraduate to the postgraduate level, as well as opportunities for interdisciplinary outreach and engagement activities with young people (Big Bang UK Young Scientists and Engineers Fair, input into British Science Association CREST projects) and adults (talks at museums and science festivals). See the website https://ancientbiotics.co.uk for full project details and team publications.

It is beneficial at this point to consider the relevance of medieval medical remedies.^3^ At the core, this research is an extension of the study of biologically active substances, and their synergies, present in natural products (such as plants) employed in so-called folk remedies or knowledge derived from traditional and historical practices, also known as ethnopharmacology. Furthermore, the methodologies from network science are useful tools to extract meaningful information from medieval ingredient datasets and to overcome the laborious task of analyzing medieval texts by hand.^4^ It should be noted that scientific investigations of the efficacy of plant-based traditional or historical medicine is well established outside of a Western context. However, for some observers, using medieval medical ingredients as inspiration for modern research may conjure disagreeable images of the most ineffectual treatments from medical history. In addition, despite a trend in publications effectively challenging the “Dark Ages” narrative, even for some scholars it is difficult to disconnect from the longstanding view of the past as a time of stagnation for science and medicine. After all, these remedies were developed under the influence of very different beliefs about health and healing, and medieval “science” was not science in the modern sense.

The aim of understanding the context and construction of remedies in extant historical texts should not be confused with making claims about past lived experiences. It is not possible to replicate medieval conditions for many reasons, including differences in soil, climate variation, the alteration of plants by human interference and evolution of species, natural variation in active ingredient composition,^5^^,^^6^ and other unknowns (such as temperatures, timings, measurements, ingredient substitutions, modifications based on patients, differences in practice) due to limitations in textual accounts. Many modern plants have been cultivated across time, and it is possible that this process may have altered their chemical signatures. It is important to be informed about genetic diversity in historical plants compared with modern plants and to refrain from drawing conclusions about historical experiences based on modern laboratory outcomes. Historical combinations of ingredients, as reported in textual sources, are a starting point to inspire thinking about potential synergies between natural products available in a modern context for application to modern needs rather than to recreate a past condition, which is not possible.

There is vibrant scholarship in using medicine of the medieval West as a vehicle to explore the “science” of the period and to understand reactions to illness and disability along with considerations of the transmission and cultural context of medical remedies (in excellent publications by scholars such as Peregrine Horden, Anne Van Arsdall, Wendy Turner, Elma Brenner, Faith Wallis, Carole Rawcliffe, Irina Metzler, Sarah Baccianti, Deborah Hayden, Siobhán Barrett, Diana Luft, and Jonathan Hsy, to cite a brief selection).^3^ The question of whether these remedies “worked” is not as frequently addressed due to the tricky and nuanced reasons mentioned above. Evaluation of historical remedies has benefited from collaboration with scientists who possess the expertise to identify plant interactions that may have present applications and to advance our understanding of how these remedies are put together in medieval written records.

In addition to expertise from medieval studies and microbiology, collaboration with data sciences, to interrogate historical datasets, has been a vital link to inform the identification of ingredients for laboratory testing. While we now know from well-supported experimental results that some of the ingredients presented in historical texts have useful biological properties, research in this area is limited by the labor-intensive burden of searching medieval texts if not assisted by algorithmic processing. In our pilot study^4^ and following projects,^3^^,^^7^ we demonstrated the potential of turning medieval medical texts into electronic datasets amenable to investigation by the methods of network analysis. This work applies algorithms from complex network science to the exploration of historical medical datasets for underlying patterns in ingredient combinations, which has a guiding influence on the direction of subsequent experimental design.^8^

In our pilot study of one fifteenth-century medical text, the network analysis identified well-understood ingredients (such as vinegar combined with medicinal plants), but, importantly, it also drew out patterns of unanticipated and understudied components, such as breast milk, frankincense, sumac, and *Aloe* spp. Likewise, our analysis of a medieval medical dataset of Old English, Middle English, Welsh, and Latin remedies, spanning the ninth to the fifteenth centuries, identified a combination of fresh nettle leaves with culinary fats and oils to treat wound infections.^3^^,^^7^ This is a curious result from the data-mining arm of the project, as these ingredients (fats and oils) do not immediately suggest efficacy from a modern microbiology perspective and would not be the primary choices selected for testing without the supporting input from dataset analysis. Furthermore, in our systematic review of nettle literature,^7^ all the studies used dry plant matter. The use of fresh nettle leaves identified in the medieval dataset is a noteworthy contrast to modern attempts to extract molecules from dried nettle material, which showed no meaningful antimicrobial efficacy. This has implications for laboratory experimental decisions, understanding of remedy construction, and for wound-dressing development. In our study, the scientists found fresh nettle leaves to be highly absorbent of liquid. The nettles had no effect on viable bacteria numbers themselves but were able to carry sufficient vinegar with them to completely eradicate the bacterial populations.^7^ Many of the historical remedies that we identified in our dataset of medieval medicines are constructed as poultices to be placed on wounds. It is possible that nettles were used at least partly for their ability to enable large volumes of medicinal liquid (like vinegar, a well-established bactericidal agent^7^) to be delivered topically to an infection site and remain securely in place. Additionally, our systematic review showed the possibility that lipophilic extracts of nettle may have antiinflammatory activity and that preparing nettles in culinary fats and oils might produce antiinflammatory extracts, although we did not directly test this ourselves.^7^

Medievalists are inclined to write complex explanations of medieval terms and the social-medical context to capture variations in interpretation, which can create challenges for scientists who are accustomed to straightforward protocols to design experiments or process data. Another example of this approach is the project Alchemies of Scent, an interdisciplinary collaboration between scientists and humanities to understand the material, cultural, and scientific context of Greco-Egyptian perfumes via replication of ancient recipes.^9^ The team uses the process of experimental replication to understand the chemistry and to fill in missing technical knowledge. In regard to their experimental process, they state the following:

We can’t know ahead of time what a correct interpretation of a recipe will look like. In fact, there may not even *be* a correct interpretation of a recipe … That’s why even though studying the text of a recipe is an important first step, we still need to try them out, testing and documenting different interpretations to see what might be going on chemically.^9^

We have found an analogous experience in our work with historical medical recipes. Medieval texts often present general recipes with the expectation that they will be modified by the practitioner performing the remedy based on many variables such as the specific characteristics of the patient (gender, age, “strength,” results of medieval uroscopy), stage of the ailment, how the condition responds to treatment, and what ingredients are available. Often, texts are silent about the technical aspects of preparing the recipe, and experimental replication can help to expose these gaps and raise relevant questions while refraining from making concrete claims about “correct interpretations.”

Our aim was thus not to make perfectly faithful recreations of medieval remedies (this would be impossible) or to draw absolute conclusions about medieval texts or practices based on outcomes in modern laboratories. Rather, we aimed to use the process of remedy reconstruction as a pathway to thinking about how medieval physicians practiced: what preparation steps are explicit or implicit in the recorded recipe, and thus what knowledge is assumed on the part of the user of a medical text? And does this preparation have any biological activity in laboratory tests that approximate its clinical use? In parallel with this, we aimed to strengthen the link between the study of historical medicine and modern scientific approaches to the discovery of natural products with potential to be developed into clinically useful formulations. We sought to understand if the preparation steps used in historical remedies that have some biological activity form an essential prerequisite to finding natural products with clinical potential. This aim depends on the recognition that, in previous studies of natural product potential, following historical recipe instructions has proved critical in observing successful activity (artemisinin^10^^,^^11^ and Bald's Eyesalve^2^^,^^12^^,^^13^ are high-profile examples, as well as the work of Larry Principe in reconstructing alchemical recipes^14^^,^^15^ and an interdisciplinary team working on the therapeutic potential of recipes in medieval Arabic medical texts^16^). It is not always easy to clearly communicate that while these parallel aims are interlinked, they are different. Thus, the team must find a balance between the limits of interpreting historical texts (and communicating such to aid the other disciplines) and the precise information required for robust modern experimental procedure.

The following discussion aims to evaluate our collaborative successes and challenges through the perspectives of the three main disciplines involved in the Ancientbiotics consortium: microbiology, data science, and medieval studies. The broad presentation of our collective experience will hopefully serve as a guide for other collaborations from the arts and humanities embarking on research that challenges conventional thinking and partnerships.

## Team building across disciplines

Relevance to multidisciplinary concerns and global issues is a current expectation for most academic research projects, particularly for humanities disciplines, and even more so for small disciplines within the humanities, such as medieval studies. Many ideas about medieval medical “science” are still to be explored, and interdisciplinary collaboration is one avenue of generating new perspectives in this area.^17^^,^^18^^,^^19^^,^^20^^,^^21^ Collaborative projects have been a driving force for creating new fields and meeting large challenges. They also provide disciplines with renewed relevance against a background environment that is always seeking to sound the death knell of irrelevance.^22^ To stay innovative, interdisciplinary teams must balance forming dynamic partnerships that meet current complex research questions and directing research aims to the future. Anticipation of future questions, and the tools needed to address them, should be part of these team relationships, which naturally seek to build on combined strengths in an additive way.^23^^,^^24^^,^^25^^,^^26^^,^^27^ In theory, this is an excellent objective for the foundation of a team: the ability to overcome the complexities of current research needs while innovating future tools. However, it is sometimes difficult for interdisciplinary teams to enact in practice because of different research perspectives and methods.^28^^,^^29^ For example, a medievalist on the Ancientbiotics team remarked to one of the science, technology, engineering, and mathematics (STEM) team members that research had been significantly delayed due to the lengthy closure of archives during COVID-19 restrictions (referring to physical libraries that hold special collections). The STEM colleague responded with confusion that archives had never closed (referring to digital preprint “archive” repositories such as bioRxiv). This is a simple example of how easily miscommunication between disciplines can result over common terms with different definitions. This error was easily corrected, but for more complex situations, time and skill are required to explain differences that may contradict automatic reflex definitions and create misunderstanding.

This is particularly the case when communicating the research to disciplines that lack training in laboratory science and expertise in understanding scientific or data concepts and when confronting gatekeeping attitudes.^30^ For instance, a popular forum from the history of medicine community generated fearful speculation about a pilot study performed by the Ancientbiotics team including a query about whether the University of Nottingham knew that its (microbiology) staff was working with pathogenic bacteria, because this was dangerous. This low level of understanding from the humanities regarding academic microbiology research in containment laboratories is surprising, but it highlights the need for more communication across the traditional arts/sciences divide. Such a perspective is not an isolated incident and has real implications when held by individuals in power with decision-making capacity for funding or hiring opportunities. The subjective experience of reviewers is another factor that can make interdisciplinary peer review difficult. One example that we have encountered is maths phobia from some humanities researchers, leading to an unwillingness to engage with projects based on complex networks analysis. A further example is a comment from a STEM reviewer questioning why the research “just could not be performed by scientists with good dictionaries,” which shows ignorance of the expertise and training required to understand and properly contextualize historical documents and historical matters in general. In contrast, there are examples of academics with an interdisciplinary background who have been instrumental in changing their field. Charlotte Roberts is one example. With a background in healthcare, she has asked questions about the conditions of care and support of people in the past and ethical implications.^31^ The partnership of Véronique Pitchon and Pierre Fechter (along with others) to examine the antimicrobial potential of metals in the medieval Arabic pharmacopeia sheds new light on overlooked historical ingredients and challenges conventional assumptions about alchemical or metal components in medieval infection remedies.^16^^,^^32^

The concept of interdisciplinarity is widely lauded as a positive attribute and has even become something of a buzzword in funding calls. However, in practical application, research that involves multiple disciplines and multiple institutions often falls through the cracks of traditional disciplinary funding, or the funder only recognizes one aspect of the collaboration.^33^ Teams may also encounter difficulties in quantifying the “value” of interdisciplinary work in the UK Research Excellence Framework and other academic assessment submissions.^34^ The Ancientbiotics collaboration has encountered no problems in obtaining support from traditional medical research funding bodies for testing historical remedies with the aim of compound discovery, but this excludes the collaboration with experts in interpreting the medical, social, and textual history. Likewise, traditional arts funding bodies may be interested in the textual or linguistic history but recoil at collaboration with sciences or maths often due to a lack of sufficient expertise to adequately review such cross-disciplinary proposals. Interdisciplinary project proposals may also require a budget that appears unusually large to reviewers or funders used to assessing single-discipline proposals. However, there are influential institutions and funding bodies with global reach, such as the Royal Society APEX Award and UK Research and Innovation, who are intentionally driving real culture change by providing a different evaluation process to truly understand the vision of these teams, to embed diversity in innovation, to remove barriers to interdisciplinary collaboration, and to capture the impact of interdisciplinary research.^35^

## Microbiology perspective of historical remedies

Communication within teams is essential, as the priorities within an interdisciplinary team are often different. For scientists, there is the temptation to focus on something that works regardless of its historical accuracy. However, this is counterproductive to the historical aims, which hope to understand and delve further into the importance of the recipe. Some practical considerations of conducting laboratory research on reconstructed historical remedies led to some fascinating conversations in our team about exactly what certain terms in historical texts meant. This was most evident with Old English plant names, which often lack an unequivocal modern translation. For example, “cropleac” occurs in many remedies and is supposed to be an *Allium* species, but there is debate about which species exactly is referred to. This raises some questions: how may we identify a “correct” species here; after all, the chemical composition of plants across the *Allium* family is not the same.^36^ Secondly, many of our existing documents from early medieval England are from the (relatively short) period of post Benedictine Reform and were largely written in southern England, and these documents are often copies of earlier manuscripts that are no longer extant. Therefore, they may reflect dialect variations of other regions or periods. Many remedies are also translations from other sources, so we need to consider that translators, too, may have had a choice of how they rendered certain species. Another question is whether the writer was actually a botanist, so would they have known the plant and, more importantly, would they have known the variants? During our APEX-funded research project on nettles, which explicitly combined humanities-led and STEM-led approaches (consideration of knowledge transmission networks plus laboratory tests of nettle preparations guided by their use in historical remedies), other communication challenges arose.

One such challenge is that medieval texts do not communicate procedures according to the research principles expected in modern laboratory experimentation; they do not contain ready protocols. For example, the specification of plant juice is a frequent occurrence in medieval nettle remedies, but what does this translate to in a laboratory context? In Middle English, juice in a medical sense can mean the actual plant juice derived from squeezing or pounding the plant matter but also the “juice” obtained by boiling the herb/plant in water or an infusion or a mixture of ground herbs and liquids.^3^^,^^37^ When preparing nettles for the experimental work in this study, by pounding the plant material with water or vinegar, the scientists observed immediately that the nettle leaves were highly absorbent: water or vinegar very quickly soaked up into the leaves, leaving no visible liquid in the mortar.^3^^,^^7^ At the beginning of the experiment, we assumed that the “nettle juice” in the medieval remedies referred to liquid extracted from pounding or squeezing the plant material; however, the practical result indicates that nettle leaves, constructed in poultice preparations, may have been favored for their ability to absorb large quantities of medicinal ingredients, like vinegar, and to deliver them securely to the treatment site. After considering our initial experimental results, Frances Watkins, medical herbalist, performed further experiments to extract nettle juice based on her practical experience of plant preparations. She was able to extract 12.5 mL juice from 50 g nettle tops (freshly harvested), which did not require straining. She suggests that medieval practitioners may have expressed the nettle juice, poured it into a glass container, and then added oil over the top as means of preservation (F. Watkins, personal communication).

Once past the biological assessment issues, our next step is to decipher the molecules behind the remedy. In most natural product research, the protocols for examining chemical qualities are based on looking at a single ingredient, such as copper or garlic, but our research is based on mixtures. With every mixture being so unique, the chemical analysis is often difficult, with no “standard” protocols in place to identify synergistic pairs or groups of natural products present in an ingredient mixture (but see, e.g., work by Nadja Cech and colleagues).^38^^,^^39^ Many standard techniques attempt to simplify natural products using fractionation, which requires the use of solvents to separate groups of molecules with similar chemical properties from the original complex mixture. This process, however, did not help us isolate one or a small number of molecules that recapitulated the activity of Bald’s Eyesalve.^40^ Having an interdisciplinary team to provide different perspectives and approaches on such problems has been invaluable in the progress of the chemical analysis conducted. However, a team that spans multiple countries will always face logistical constraints that slow progress. For example, meetings must be arranged in the future (no quick chats), samples require sending and laborious safety checks, and equipment and time must be managed across a larger number of people.

We must also consider that the activity of the remedy may vary depending on the physiological state of the microbes against which it is employed. The microbiologists forming the STEM nucleus of the Ancientbiotics consortium all work on bacteria that form multicellular aggregates called biofilms—this is the natural state for bacteria in many infections, including eye, skin, and soft tissue infections. So, when testing Bald’s Eyesalve, our team recognized that the probable indication in the original remedy (an eye infection) and potential other applications of the remedy if effective (wound infections) are caused by biofilms. Thus, we tested the remedy against bacteria grown as aggregated biofilms in a solid model of soft tissue. This revealed that all ingredients in the original remedy were required to make an effective preparation. A very different result can be obtained if bacteria are grown as free-swimming single cells in liquid broth (planktonic culture)—which is the basis of most standard research and diagnostic antibiotic activity testing. In this case, testing would have, and did,^41^ incorrectly attribute all of the activity of the remedy to a single molecule present in one ingredient. This molecule is present in the remedy at concentrations sufficient to kill planktonic cultures but not to kill the same bacteria grown as biofilms. Thus planktonic testing misses important synergistic interactions between ingredients in the recipe that are necessary to kill biofilms.^2^^,^^12^^,^^13^^,^^40^^,^^42^ This is a strong case for incorporating interdisciplinary aims to guide scientific practice and improve on standard protocols.

A final example is the issue of variation in plant natural products mentioned above. Research within natural products is well understood to be impacted by the biological variation found in plants.^43^^,^^44^^,^^45^^,^^46^^,^^47^^,^^48^ In our case, these problems are further exacerbated by the unknowns of historical recipes, such as the inexact measurements, lack of specific storage instructions, and ambiguities in translations, all of which can significantly alter the chemical composition, with or without altering biological activity. These combined make finding patterns that are important rather than incidental exceptionally difficult.

## Data-mining approach to historical data

Similar to the chemical analysis, when constructing medieval datasets for analysis using modern computational tools, there are several challenges that require creative or unconventional solutions. These challenges include spelling and language variation, multiple synonyms for the same ingredient, terms with many possible interpretations or ambiguous definitions, decisions about translating terms, inclusion and exclusion criteria for presenting remedies in dataset form, decisions around eliminating noise in the dataset, and the variation within the modern system of botanical binomial nomenclature. Constructing and standardizing (or cleaning) medieval datasets and subsequent interrogation by algorithms is not a uniform methodology. In documenting our approaches (in our published studies), we intend to be transparent about the challenges and choices and to add to ongoing conversations about structuring “fuzzy” data in the current digital space.^4^

Indeed, these ambiguities, and the decisions taken to solve them, have numerous implications that also affect the algorithmic part of the data analysis. As a central illustrative example, consider the occurrence of multiple terms referring to different parts of the same plant. The decision that must be made in such situations is whether to consider each term as a separate ingredient or instead to collate them into a single meta-element. The choice is less straightforward than it might appear at a first glance. In fact, maintaining the separation between closely related ingredients certainly increases the resolution of the resulting dataset. If the goal is to build a network of ingredients, this will look appealing, as the final structure will have a higher number of nodes, and, therefore, algorithms rooted in statistical physics will provide better results, minimizing finite-size effects. However, this is only true if one is operating on a very large number of recipes and each of the individual elements appears a significant number of times. If, instead, these conditions are not met, the strength of the edges involving these ingredients in the final network is necessarily low. As a consequence, the uncertainty on the significance of these connections increases as the data become more noisy. One could then be tempted to always take the opposite approach and collate the ingredients into meta-nodes whenever possible. This is, in fact, quite a valid choice when the number of recipes or that of the individual ingredients is small. In such cases, the noise has a more prominent effect, and it is therefore more important to reduce it. However, these situations are also those when the final network tends to have a smaller size, of the order of the few tens of nodes. Thus, it also becomes important not to reduce this size even further, beyond what is strictly needed, and to aim for a judicious application of these criteria.

Deciding how to treat closely related ingredients is also a question that illustrates well the added benefits of interdisciplinarity. The reason is that, regardless of algorithmic considerations, it does not make sense to consider multiple ingredients to be the same if their chemical contents have substantial differences. For instance, some remedies may refer separately to the seed and the aril of the nutmeg plant (*Myristica fragrans*) or to the leaves and the fruit of the aubergine (*Solanum melongena*). However, while the former two have effectively the same content of active compounds, albeit with a slightly different aromatic balance, as many home bakers will know, the latter pair differs significantly to the point that the aubergine leaves are toxic, unlike the fruit, which is edible. Team members with an expertise in plant biology can provide major help in recognizing such cases. At the same time, their contribution cannot be isolated from that of linguists and historians, who can interpret the original text and identify the meaning of the different terms. This means that the work has to be a choral effort, with a strong emphasis on frequent, if not continuous, communication.

By itself, this can bring its own challenges due to the different assumptions that people in different fields may subconsciously make. For example, a data scientist may consider a network with 30 nodes as being small. Indeed, such a size is fairly close to the limit of what one could meaningfully analyze via community detection. However, at the same time, researchers from other areas may think that having 30 different ingredients, all potentially active, constitutes a wealth of data. Fortunately, such difficulties can be easily overcome by a process of “explaining the obvious,” motivated by the fact that some pieces of information may, after all, not be obvious at all to people outside a specific field of research.

## Challenges of medieval ingredients in modern research

Many of the remedies use ingredients from the kitchen or farm, such as butter and lard. Others use alcohol, such as beer.^49^ What is important is that these foodstuffs were not made in a commercial or standardized production. Butter may have been more or less salted, and ale was produced in small batches, usually by women in domestic environments. The brewsters may have used oats instead of barley; they may have added herbs or fruit. The Old English word “beor” may be a cognate to modern English beer but is very different from it since it could be brewed with honey and fruits and, according to Hagen, was a sweeter beverage than what we are used to.^50^ Each of these components may thus introduce a different chemistry and one which is very difficult to guess. To add complexity, “beor” in medical remedies comes in various versions: from “niwe” (new) and “leoht” (light) to “strang” (strong)—and the exact composition is therefore largely guesswork. Similarly, “ealu,” which is the word for “ale,” can have a wider range of meaning than just ale, and the word is also qualified in remedies: “hlutor” (pure) to “willisc” (Welsh) ale. There seems to be a great variety of different forms, which clearly had meaning for the early medieval physician but take some guesswork today.

It is reasonable to express disclaimers and nuances related to the many possible interpretations of historical terms and complexities surrounding transmission of medical material. As mentioned in the introduction, historical texts often do not provide the information necessary to translate to an experiment according to modern expectations. Our systematic review and experimental work on nettles led to questions about extraction temperature and preparation time, which may impact bioactive ingredients and chemical composition. Searching the microbiological literature for brewing, stewing, incubation, or decoction times of natural products offered few examples for determining the most effective temperatures and preparation times. Often, the medieval remedies are silent about these specifics or provide generic guidelines, according to modern eyes, due to a lack of standardization and instrumentation to report such things as well as an expectation that the reader will understand the methodology and will be able to fill in the missing information from their own experience. This is similar to how a modern culinary recipe may specify “make a roux” or “prepare the pastry dough” without further instructions. This situation is addressed directly by a fifteenth-century Middle English translation of the influential “Lilium medicinae” regarding the lack of specific measurements for ingredients. The doctor is advised to “work according to his craft and teaching” determined by the specific situation of his patient.^51^ Also, some diseases and treatments carry so many possibilities that the author would prefer to leave the judgment to the physician in charge rather than to set down specific quantities or directions.^52^ The author assumes that the reader would already be familiar with the methodology and could make their own decisions about the recipes: “I resign the manner of working to him [the doctor] … and the proportion and the giving thereof.”^53^ This presents a very different scenario for us than for researchers working on living ethnobotanical traditions, where it is possible to interview real practitioners and end users of herbal and other natural preparations.^54^

## Concluding thoughts

Our interdisciplinary investigation into the multicomponent nature of historical remedies has challenged how drug discovery is approached and opened a research avenue between the arts and sciences. The results from our preliminary work speak to larger questions regarding the historical construction of antimicrobial ingredients and the application of our results to the search for novel antimicrobial drugs. We must be mindful, in this early stage, in interpreting and extrapolating the results of algorithmic interrogation and laboratory assays performed on historical ingredient combinations; however, further applications and replications of this methodology will add to this initial foundation.

## Biography

**Erin Connelly** is an assistant professor (research) in the School of Life Sciences, University of Warwick (UK), supported by a UK Research and Innovation Future Leaders Fellowship. **Christina Lee** is an associate professor in the School of English, University of Nottingham (UK). **Jessica Furner-Pardoe** is an early career research fellow collaborating between University of Warwick and UKHSA (UK). **Charo I. del Genio** is a Senior Lecturer in Statistical Physics in the Centre for Fluid and Complex Systems, Coventry University (UK). **Freya Harrison** is a reader in the School of Life Sciences, University of Warwick (UK). Please visit https://ancientbiotics.co.uk for further information.
